# Parasite specific 7SL-derived small RNA is an effective target for diagnosis of active trypanosomiasis infection

**DOI:** 10.1371/journal.pntd.0007189

**Published:** 2019-02-19

**Authors:** Stephen M. Chiweshe, Pieter C. Steketee, Siddharth Jayaraman, Edith Paxton, Kyriaki Neophytou, Heidi Erasmus, Michel Labuschagne, Anneli Cooper, Annette MacLeod, Finn E. Grey, Liam J. Morrison

**Affiliations:** 1 The Roslin Institute, Royal (Dick) School of Veterinary Studies, University of Edinburgh, Easter Bush, Midlothian, United Kingdom; 2 Institute of Immunology and Infection Research, School of Biological Sciences, University of Edinburgh, Edinburgh, United Kingdom; 3 Clinvet Research Innovation, Uitzich Road, Bainsvlei, Bloemfontein, South Africa; 4 Wellcome Centre for Molecular Parasitology, Institute of Biodiversity, Animal Health and Comparative Medicine, College of Medical, Veterinary & Life Sciences, Bearsden Road, University of Glasgow, Glasgow, United Kingdom; Universiteit Antwerpen, BELGIUM

## Abstract

Human and animal African trypanosomiasis (HAT & AAT, respectively) remain a significant health and economic issue across much of sub-Saharan Africa. Effective control of AAT and potential eradication of HAT requires affordable, sensitive and specific diagnostic tests that can be used in the field. Small RNAs in the blood or serum are attractive disease biomarkers due to their stability, accessibility and available technologies for detection. Using RNAseq, we have identified a trypanosome specific small RNA to be present at high levels in the serum of infected cattle. The small RNA is derived from the non-coding 7SL RNA of the peptide signal recognition particle and is detected in the serum of infected cattle at significantly higher levels than in the parasite, suggesting active processing and secretion. We show effective detection of the small RNA in the serum of infected cattle using a custom RT-qPCR assay. Strikingly, the RNA can be detected before microscopy detection of parasitaemia in the blood, and it can also be detected during remission periods of infection when no parasitaemia is detectable by microscopy. However, RNA levels drop following treatment with trypanocides, demonstrating accurate prediction of active infection. While the small RNA sequence is conserved between different species of trypanosome, nucleotide differences within the sequence allow generation of highly specific assays that can distinguish between infections with *Trypanosoma brucei*, *Trypanosoma congolense* and *Trypanosoma vivax*. Finally, we demonstrate effective detection of the small RNA directly from serum, without the need for pre-processing, with a single step RT-qPCR assay. Our findings identify a species-specific trypanosome small RNA that can be detected at high levels in the serum of cattle with active parasite infections. This provides the basis for the development of a cheap, non-invasive and highly effective diagnostic test for trypanosomiasis.

## Author summary

African trypanosomes cause significant disease in humans and animals across sub-Saharan Africa. For both human and animal infections diagnostics that can accurately identify an active infection are lacking–this is particularly the case in animal disease where most diagnosis is based upon clinical signs, which is not a specific or sensitive means of detecting infection. There is therefore a significant unmet need for a pathogen marker of active infection that accurately indicates whether an animal or human is currently infected. Through analysing the blood of cattle infected with trypanosomes, we identified a short sequence of RNA that was present at very high levels. This small RNA derives from the trypanosome genome, and we could identify its presence in the genome of all three species that are responsible for human and animal disease. We were able to design species-specific tests, and showed that in samples from infected animals the assays were more sensitive than the traditional microscope-based detection, importantly the signal disappeared relatively quickly after successful treatment, and when treatment failed, the assay was able to accurately identify when infection persisted. We also demonstrated that the causative agent of human trypanosomiasis secretes the marker at similar levels to that seen in the animal-infective trypanosomes. Therefore, we have discovered a marker of trypanosome infection that is present at high levels in the blood of infected animals, disappears quickly upon successful treatment, but is effective at detecting instances of unsuccessful treatment and persistent infection. This represents a potentially powerful diagnostic tool for human and animal trypanosomiasis.

## Introduction

African trypanosomes, vector borne protozoa transmitted by tsetse flies (*Glossina* species), cause Human African Trypanosomiasis (HAT) and Animal African Trypanosomiasis (AAT) across sub-Saharan Africa. AAT, caused by *Trypanosoma congolense*, *Trypanosoma vivax* and *Trypanosoma brucei*, infects approximately 70 million and kills 3 million cattle per year, and is one of the most significant infectious disease constraints upon agriculture in the region [1]. HAT is caused by two variants of *T*. *brucei*, *T*. *b*. *gambiense* and *T*. *b*. *rhodesiense*, and in recent years the impact of this disease has been significantly reduced through active case detection, with <3,000 cases reported in 2015, down from ~50,000 in 2000 [2, 3]. However, new and improved tools are required for both diseases; for AAT as a tool to begin to reduce the current significant infection burden, and for HAT to facilitate the delivery of the WHO aim of HAT elimination by 2030 [4, 5].

The ability to diagnose active infections is currently still a significant challenge for both AAT and HAT. While there have been substantial efforts to develop new effective diagnostics for HAT, currently the gold standards remain microscopy (with methods that concentrate–e.g. microhaematocrit centrifugation, quantitative buffy coat or mini anion exchange centrifuge technique (mAECT) [6–9]–all providing increased sensitivity) and the card agglutination test (CATT)–an antibody agglutination test based upon several VSGs expressed by *T*. *b*. *gambiense* [10] (for the latter assay there has also been recent adaptation to a rapid diagnostic test platform [11, 12]). While some molecular tests (e.g. loop mediated isothermal amplification–LAMP) have demonstrated promise [13], and for LAMP this has included the development of field-applicable kits, these have not been widely utilised in the field [14]. Tests based on antibody and DNA have their well-recognised limitations (differentiating between exposure and infection for the former and the potential for DNA persistence following treatment, as well as contamination, for the latter), and a test that enabled sensitive and specific detection of active infection would be a significant advance. For AAT, diagnosis is still largely symptomatic with inherent non-specificity given lack of pathognomonic clinical signs, and occasionally microscopy may be employed [15]. Investment in development of diagnostics for AAT is increasing, with recent efforts defining antibody-based capture techniques for antigens that have been described as conserved and highly expressed [16–18]. Indeed, this approach has resulted in the first commercial diagnostic being brought to market in 2017 (VerY Diag, CEVA). Therefore, available methods for both HAT and AAT have their limitations–the requirement for a test that enables detection of active infection remains–both for potential utility in the field and to improve, for example, accurate assessment of clinical efficacy of drugs and vaccines (increasing areas of interest for AAT). An ideal marker for active infection is a pathogen-derived molecule that is present in high enough levels in infected animals/patients to enable sensitive detection, has properties that enable assignment to pathogen and species to a high level of confidence, and, additionally, reduces in levels quickly following removal of the pathogen (e.g. by chemotherapy).

Small RNAs have received much interest as potentially useful diagnostic biomarkers, particularly in human medicine and cancers [19]. This is due to higher expression of particular small RNAs (e.g. microRNAs [miRNAs]) in cancer cells. In these cases, diagnosis requires confirmation of higher levels of the small RNA species in comparison to non-affected cells/tissues. For application to pathogens in contrast, the test would aim to identify the binary presence or absence of a pathogen marker, a much simpler threshold to define. Trypanosomes produce multiple small RNAs (although do not produce miRNAs) and in the best-characterised species, *T*. *brucei*, there has been description of the small RNAome [20]. The *T*. *brucei* genome includes identification of small RNA encoding loci, including rRNA, snoRNA, tRNA and siRNA [21, 22] (albeit only a proportion of these have been functionally validated). In addition, several reports have outlined the RNA species secreted/excreted in the form of vesicles by related trypanosomatids such as *Trypanosoma cruzi* and *Leishmania major* [23, 24]. There is less information for *T*. *congolense* and *T*. *vivax*, although both species have annotated genomes available with predicted small RNA-encoding genes [21, 25].

In the current study we describe a trypanosome small RNA species that is present in the serum of infected animals at high levels. This small RNA is a 26-nucleotide segment of the 7SL long non-coding RNA (‘7SL RNA’); the latter is usually described as a cytoplasmic non-coding RNA that is part of the signal recognition particle (SRP) involved in protein translocation across cell membranes. The 7SL-derived small RNA (hereafter termed ‘7SL-sRNA’) sequences are species-specific, enabling the design of tests that differentiate between *T*. *congolense*, *T*. *vivax* and *T*. *brucei*. The 7SL-sRNA is present at high levels in infected animals (equivalent to levels of highly expressed bovine miRNAs), enabling robust detection both before detection by microscopy and during periods of infection with subpatent parasitaemia. Importantly, following post-curative treatment the levels of the 7SL-sRNA drops to undetectable levels. Therefore, we believe that the 7SL-sRNA represents a suitably sensitive and specific marker for detection of active infection in trypanosomes, with potential utility for both HAT and AAT.

## Materials and methods

### Ethics statement

Animal experiments were carried out at the Roslin Institute, University of Edinburgh under the auspices of Home Office Project License number 60/4394. Studies were approved by the Roslin Institute Animal Welfare and Ethical Review Board (study numbers L172 and L223). Care and maintenance of animals complied with University regulations and the Animals (Scientific Procedures) Act (1986; revised 2013). Protocol plans for studies carried out at Clinvet were submitted to the Institutional Animal Care and Use Committee (IACUC), which issued certificates of approval. The protocol was designed to allow the use of the study animals in compliance with the Clinvet policy on the ethical use of animals, using the most recent version South African National Standard (SANS) 10386 (The care and use of animals for scientific purposes). Approved study numbers were CV 15/192 and CV 16/306.

### *In vivo* infections

Samples from infected animals derive from two sources; (i) experimental infections carried out at the Roslin Institute (*T*. *brucei* and *T*. *congolense*) and (ii) experimental infections carried out as part of candidate drug testing by GALVmed/Clinvet (*T*. *congolense* and *T*. *vivax*).

- Experimental infections of cattle (post-weaning male Holstein-Friesian cattle approximately 4–6 months of age; n = 4 per trypanosome species) were carried out in vector proof containment at the Roslin Institute. 1 x 10^6^ trypanosomes (*T*. *brucei* AnTat 1.1 or *T*. *congolense* IL3000) were inoculated intravenously via the jugular vein, and infections followed for 28 days. Parasitaemia was measured every two days in jugular blood samples by the quantitative buffy coat technique [26]. Serum or plasma was also prepared at each sampling timepoint; for plasma, blood was centrifuged at 1500 x *g* for 15 minutes at room temperature and supernatant (plasma) was removed.
- Samples were received (GALVmed/Clinvet) from *in vivo* studies in groups of cattle that aimed to test clinical efficacy of a candidate trypanocidal drug. Experimental animals (Holstein-Friesian, male and female, 2 months post-weaning and at least 4 months of age) were infected by intravenous injection via the jugular vein, 21 cattle with *T*. *vivax* (STIB 719) and 21 cattle with *T*. *congolense* (KONT 2/133), with approximately 1 x 10^5^ viable parasites in fresh cow blood. Animals were divided into control (3 animals) and experimental groups (18 animals; 3 groups of 6). Upon reaching first peak of parasitaemia, the control group was treated with saline and the 3 sample groups of experimental animals were treated with different dosages of the candidate trypanocide drug, both administered intramuscularly. A rescue treatment was administered for both control and experimental animals when parasitaemia persisted for 7 consecutive days, or clinical signs warranted intervention, in the form of isometamidium chloride (1 mg/kg) or diminazene aceturate (7 mg/kg). Infection levels were determined by microscopy every 2 to 3 days [27] and plasma samples were collected for RNA extraction and 7SL-sRNA determination at approximately weekly intervals. Plasma was prepared by centrifuging blood at 1800 x *g* for 10 minutes at room temperature and removing supernatant (plasma).

### *In vitro* culture

*T*. *congolense* IL3000 BSF parasites were cultured in TcBSF3 medium [28] supplemented with 20% adult goat serum (Gibco), 0.12 mM 2-mercaptoethanol and penicillin/streptomycin and incubated at 34°C, 5% CO_2_. Cells were routinely passaged and maintained at a density between 5 × 10^4^ cells/mL and 3 × 10^6^ cells/mL, unless stated otherwise.

*T*. *brucei* Lister 427 cells were cultured in HMI-11 [29] supplemented with 10% FBS, 0.2 mM 2-mercaptoethanol and penicillin/streptomycin, and maintained at 37°C, 5% CO_2_. Cells were maintained between 2 × 10^4^ cells/mL and 2 × 10^6^ cells/mL. Bloodstream forms of group 1 *T*. *b*. *gambiense* strain ELIANE were cultured in HMI-9 supplemented with 20% serum plus, as previously described [30].

### RNA extraction

RNA extractions were conducted using the TRIzol LS reagent (Invitrogen) following the manufacturer’s instructions. 250 μL of starting material in the form of serum/plasma for *in vivo* experimental infections carried out at the Roslin Institute (*T*. *brucei* and *T*. *congolense*) was used; where the sample was less than 250 μL, distilled water was added to make up the volume. For *in vivo* samples received from GALVmed/Clinvet, RNA was extracted from 125 μL of plasma with distilled water added to make up the volume to 250 μL. In cases where *in vitro* culture supernatants were used for RNA extraction, 500 μL supernatant was centrifuged at 2,000 × *g* for 10 minutes to remove cells from the medium. Subsequently, 250 μL supernatant was used for downstream experiments.

### RNA deep sequencing

Libraries were prepared using the TruSeq Small RNA library preparation kit (Illumina) with 10 μL total RNA as starting material (quantity of total RNA for each sample: uninfected serum– 39ng, infected sample 1 – 230ng, infected sample 2 – 270ng, parasite cell pellet– 2.1μg). Samples were enriched using 15 cycles of PCR and library products of 145–160 bp were gel purified, quantified and pooled for sequencing. The library pool was sequenced using a HiSeq 2500 with 50-base single end reads and V4 chemistry.

### RT-qPCR

A species-specific 7SL-derived small RNA stem loop primer-probe detection assay was optimised, using custom primer and probe mixes made by Life Technologies, based on specific sequences (Custom TaqMan Small RNA assay, cat. number: 4398989 [assay IDs *T*. *brucei*: CTFVKNM; *T*. *congolense*: CTRWEM9; *T*. *vivax*: CTDJXGZ]). Reverse transcription was carried out using a commercial cDNA Reverse Transcription Kit (Applied Biosciences, cat. number: 4368814), replacing the random primers with the aforementioned TaqMan assay primer. Typically, 100 ng RNA was used per 15 μL reaction. The following thermocycling conditions were applied for the RT reaction: 16°C for 30 minutes, 42°C for 30 minutes and 85°C for 5 minutes to inactivate the reverse transcriptase.

*In vivo* RNA samples from the GALVmed/Clinvet trial were isolated from plasma derived from heparinised blood and therefore required 2 units of *Bacteroides* Heparinase 1 (New England BioLabs, cat. number: P0735) per RT reaction.

Subsequent to the RT reaction, a qPCR was performed using a commercial kit (TaqMan universal PCR master mix, Thermo, cat. number: 4304437), according to manufacturer’s instructions. At this stage, 1 μL custom prime-probe was also added to the qPCR reaction, along with 1.5 μL RT reaction. The qPCR cycling profile was as follows: 50°C for 2 minutes, 95°C for 10 minutes and 40 cycles of 95°C for 15 seconds and a probe detection step of 60°C for 1 minute.

When serum was used as a substrate for RT-qPCR, samples were heat treated at 65°C for 15 minutes and 6 μL of the serum used per RT reaction. Single step RT-PCR reactions were performed according to manufacturer’s guidelines (TaqMan RNA-to-Ct 1-Step Kit, Life Technologies, cat. number: 4392653). Single step RT-PCR reactions were performed using the TaqMan small RNA assay primer and primer-probe mixes mentioned previously.

### Bioinformatics

Raw RNA deep-sequencing data were subject to quality control using FastQC (v0.11.5) [31]. Adapter sequences were then removed from the reads, and data was filtered for read length between 20 and 39 base pairs using cutadapt v1.7.1 (parameters: “–a TGGAATTCTCGGGTGCCAAGG–m 20 –M 39”) [32] Reads were subsequently aligned to the *T*. *congolense* IL3000 genome (TriTrypDB, v9.0), using Novoalign (v3.02.12, Novocraft Technologies), with the following parameters: “–l 20 –t 30 –h 60 –m–o SAM”. To quantify the alignments, the python tool HTSeq-count was used with default parameters [33] with the *T*. *congolense* IL3000 transcript reference file in .gff format downloaded from TriTrypDB (v9.0) [21]. Data was normalised by calculating reads per million (RPM): all read counts in a sample were first summed and the sum divided by 1 million to generate a “per million” scaling factor. Read counts were subsequently divided by this scaling factor to generate the RPM value for each gene. Raw and processed data is available through GEO accession number GSE122858.

## Results

### Small RNA derived from the non-coding 7SL is detected at high levels in the serum of infected cattle

Small RNAs in blood represent attractive diagnostic biomarkers as they tend to be relatively stable, are easily accessible, and sensitive technologies exist for direct detection from serum samples. To test whether trypanosomes secrete or excrete small RNAs during *in vivo* infections, total RNA was extracted using Trizol LS from serum samples obtained from two cattle experimentally infected with the livestock trypanosome *T*. *congolense* (samples taken at day 19 post-infection, parasitaemia at time of isolation approximately 5 x 10^6^ /mL), serum from an uninfected control cow, and an *in vitro*-derived *T*. *congolense* cell pellet (approximately 4 x 10^7^ cells). The RNA was submitted for small RNA deep sequencing, selecting for RNAs between 20- and 39-bp long. The resulting reads were aligned to the *T*. *congolense* genome (TriTrypDB v9.0) using novoalign with strict parameters (one mismatch per read and a homopolymer filter score of 60; normalised results and alignment statistics, are available in S1 Table and S2 Table).

A total of 15,645,557 and 16,770,619 reads were obtained for the two samples from infected cattle after filtering for read length between 20-bp and 39-bp. Of these, 4.2% (654,025 reads) and 1.3% (218,788 reads) were uniquely mapped to the *T*. *congolense* genome. 6,290,490 reads were obtained from the uninfected cattle sample, with only 0.03% (1,804 reads) aligning uniquely to the *T*. *congolense* genome. Reads were also aligned to a bovine genome (*Bos taurus*, UMD [34] (S2 Table). These results indicated that 87.4%, 87.3% and 78.6% of reads from the first infected, second infected and uninfected cattle samples, respectively, aligned to the bovine genome (including both uniquely aligned and multimapped reads).

A total of 9,439,764 reads were generated from RNAseq of the *T*. *congolense* cell pellet sample, with 11.5% (1,084,005 reads) and 58.9% (5,557,663 reads) unique and multimapped reads aligning to the *T*. *congolense* genome, respectively. The relatively high number of unmapped reads (2,787,320; 29.5%) in this sample with respect to the *T*. *congolense* genome is explained by the comparatively incomplete assembly and annotation of the reference *T*. *congolense* genome when compared to, for example, the genome of *T*. *brucei*.

**Fig 1:**
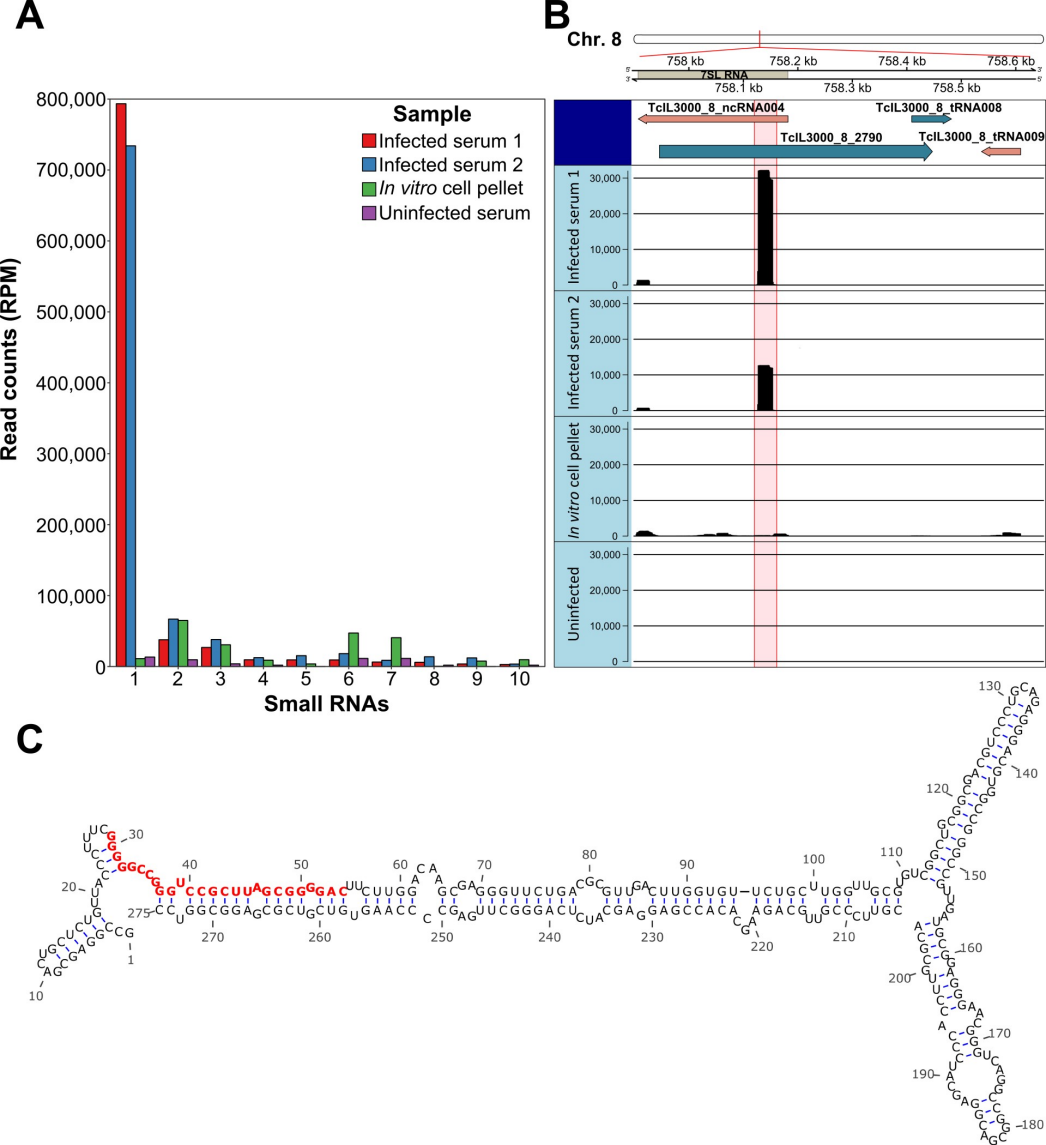
RNA-sequencing of serum isolated from *T*. *congolense*-infected cattle reveals a parasite-specific 7SL-derived small RNA. A) Read counts normalised by RPM of the ten most abundant small RNAs detected in *T*. *congolense*-infected serum (Gene IDs: 1 –TcIL3000_8_ncRNA004; 2 –TcIL3000_10_12320; 3 –TcIL3000_0_34310; 4 –TcIL3000_0_tRNA019; 5 –TcIL3000_10_tRNA006; 6 –TcIL3000_10_12310; 7 –TcIL3000_0_25440; 8 –TcIL3000_10_tRNA003; 9 –TcIL3000_10_tRNA002; 10 –TcIL3000_0_14280). B) Visualisation of the location of the 7SL-sRNA identified at high abundance in infected serum. The panels below show read alignments of the 26-bp sequence in two serum samples from infected cattle, as well as read count from an *in vitro*-derived *T*. *congolense* cell pellet and an uninfected serum control, in the form of histograms. Also visible is a smaller peak corresponding to a potential passenger strand. C) The mfold web server was used to generate the predicted secondary structure of the *T*. *congolense* 7SL RNA. The 7SL-sRNA is highlighted in red.

**Fig 2:**
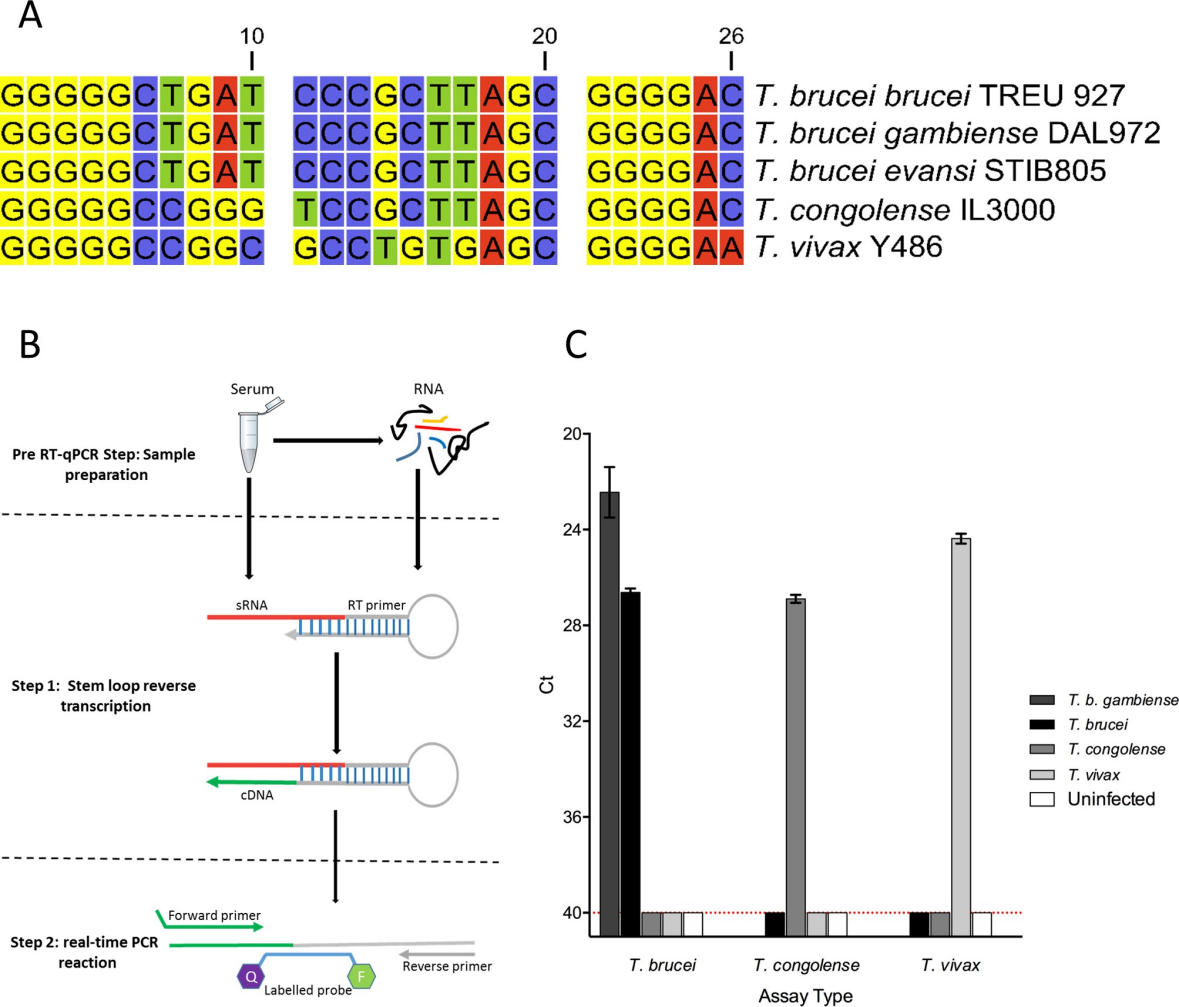
7SL-sRNA sequence enables differentiation between animal-infective trypanosome species. A) The 26-bp 7SL-sRNA sequence identified in *T*. *congolense* serum was aligned to that from other trypanosomatids, revealing species-specific differences that were flanked by conserved guanosine repeats. The polymorphisms were utilised to design species-specific Taqman RT-qPCR assays. B) Schematic representation of the Taqman RT-qPCR assay, a 2 step based assay that makes use of step-loop primers as previously described in [56]. C) Assays generated for the detection of *T*. *b*. *brucei*, *T*. *vivax* and *T*. *congolense* were applied to RNA samples extracted from plasma from infected animals, adjudged to exhibit similar parasitaemia scores. In each case, only the species for which the assay was designed was detected by RT-qPCR and were statistically significant (alpha = 0.05) as determined by Wilcoxon Signed Rank test, P value (two tailed) <0.0001 and n = 4. Red dotted line indicates limit of detection. The *T*. *brucei* assay was also tested on RNA extracted from *T*. *b*. *gambiense* culture supernatants (n = 3, average cell density of 1.56 x 10^6^ cells/mL).

Subsequent analysis showed that the majority of mapped reads in the uninfected cattle sample that mapped to the *T*. *congolense* genome aligned to ribosomal RNA (rRNA) loci, the sequences of which are known to be deeply conserved in eukaryotes [35]. For this reason, rRNA alignments were omitted from downstream analyses as a data filtering step as they are therefore unlikely to be useful molecular diagnostic targets. Read counts from annotated regions of the *T*. *congolense* genome were generated using HTSeq-count, resulting in total read counts mapping uniquely to annotated features of 42,302 and 18,230 for the two infected samples, 322,385 for the pellet sample and 524 for the uninfected sample. Read counts were normalised for library depth (reads per million; RPM). Strikingly, after eliminating reads associated with rRNA loci, the majority of reads from both infected serum samples originated from one specific 26-bp sequence (Fig 1; normalised dataset in S1 Table). Indeed, there was a substantial difference between the abundance of this small RNA and the next most abundant small RNA observed in infected serum, as well as uninfected and cell pellet controls (Fig 1A, detailed in Table 1). The reduced levels of the small RNA in the cell pellet sample relative to the serum samples and subsequent analysis of culture supernatant (see below & Fig 2), suggests that the RNA species is rapidly secreted/excreted from the cell post-processing. Further analyses indicated that the sRNA uniquely mapped to a single copy locus on chromosome 8 that comprised part of the 275-bp 7SL RNA gene (Signal Recognition Particle (SRP) RNA, *T*. *congolense* Gene ID: TcIL3000_8_ncRNA004; *T*. *brucei* Gene ID: Tb927.8.2861) (Fig 1B), and is henceforth referred to as “7SL-sRNA”. The full secondary structure of the 7SL RNA is shown in Fig 1C, with 7SL-sRNA highlighted in red. Notably, a sequence corresponding to the 7SL-sRNA complementary strand was also detected in the RNAseq data, although at approximately ten-fold lower abundance, suggesting the existence of a passenger strand following processing of the 7SL-sRNA (Fig 1B). There were no sequences corresponding to the host 7SL RNA detected, suggesting that generation of a small RNA from the 7SL RNA is specific to trypanosomes and not a general feature of 7SL RNA processing.

**Table 1:** Normalised read counts (RPM) of the 10 most abundant small RNAs detected in *T*. *congolense*-infected host serum (excluding rRNA).

Gene ID	Infected serum 1	Infected serum 2	*In vitro* cell pellet	Uninfected serum
TcIL3000_8_ncRNA004-1	793,414.02	734,064.73	11,114.04	13,358.78
TcIL3000_10_12320–2	37,752.35	66,867.80	64,959.60	9,541.98
TcIL3000_0_34310–1	26,901.80	38,069.12	30,655.89	3,816.79
TcIL3000_0_tRNA019-1	9,503.10	12,506.86	8,924.11	1,908.40
TcIL3000_10_tRNA006-1	9,384.90	15,304.44	3,694.34	0.00
TcIL3000_10_12310–1	9,290.34	18,211.74	47,238.55	11,450.38
TcIL3000_0_25440–1	6,311.76	8,776.74	40,656.36	11,450.38
TcIL3000_10_tRNA003-1	5,957.17	13,768.51	409.45	1,908.40
TcIL3000_10_tRNA002-1	3,616.85	12,177.73	7,683.36	0.00
TcIL3000_0_14280–1	2,836.75	3,455.84	9,665.46	1,908.40

### RT-qPCR assay targeting trypanosome 7SL small RNA is highly sensitive and species-specific

The 7SL-sRNA sequence of *T*. *congolense* was aligned to the genome assemblies of several species of African trypanosome to determine whether related trypanosome species encode for the 7SL-sRNA species. Sequences corresponding to the 7SL-sRNA were clearly identifiable in the genomes of all related trypanosome species examined, suggesting expression of 7SL-sRNA may be a common feature of African trypanosomes, and indeed, related trypanosomatids. Whilst no sequence variation was observed across any of the *T*. *brucei* subspecies (specifically *T*. *b*. *brucei*, *T*. *b*. *gambiense*, *T*. *b*. *rhodesiense* and *T*. *b*. *evansi*), there were several nucleotide polymorphisms relative to the *T*. *brucei* sequence in both the *T*. *vivax* and *T*. *congolense* sequences, raising the possibility that specific assays could be designed to distinguish between the three primary livestock trypanosome pathogens (Fig 2A). To investigate this further, custom-designed primers were developed using existing stem-loop technology for each individual species and RT-qPCR experiments performed (Fig 2B). Each primer set was applied to RNA extracted from serum samples from cattle experimentally infected with each species to test for cross-reactivity. Results show that sequence divergence of the 7SL-sRNA is sufficient to enable the design of RT-qPCR assays that differentiate between *T*. *vivax*, *T*. *congolense* and *T*. *brucei* with no detectable cross-reactivity (Fig 2C). Importantly, when applied to supernatants derived from the human-infective *T*. *b*. *gambiense* (ELIANE strain) the *T*. *brucei* RT-qPCR assay resulted in positive detection of the 7SL-sRNA, highlighting the potential of the sRNA for diagnostics in human disease (Fig 2C).

### Monitoring *in vitro* 7SL-sRNA excretion/secretion

**Fig 3:**
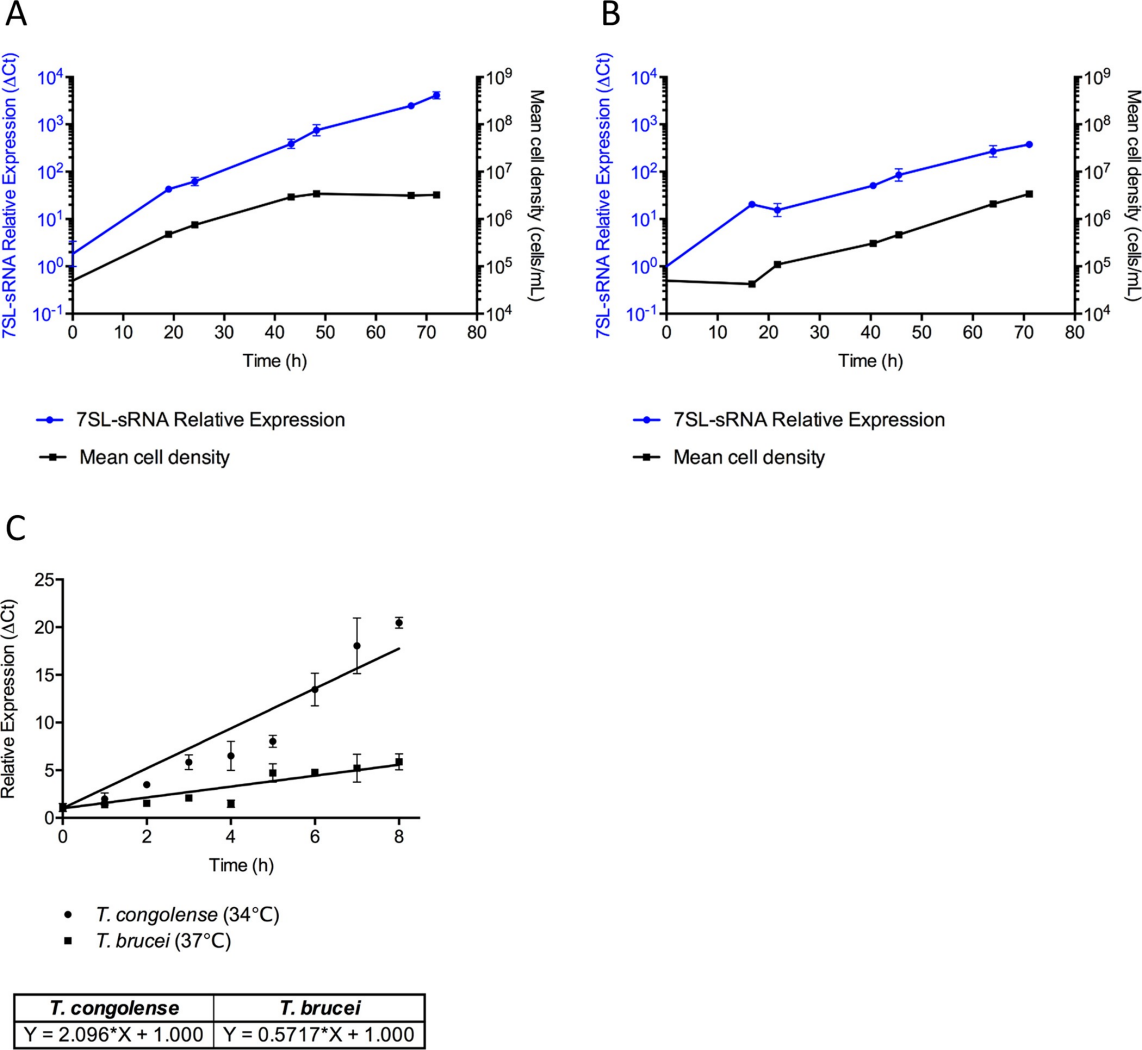
7SL-sRNA abundance is correlated to cell density in cultured trypanosome supernatants. Cell density of *in vitro* cultures of *T*. *brucei* (A) and *T*. *congolense* (B) was monitored over time and supernatant samples were simultaneously isolated for 7SL-sRNA detection (n = 2 per time point). (C) Relative levels of the 7SL-sRNA, normalized to the 0 hour time-point control, were observed to increase as cell density increased; statistical significance of correlation between cell density and relative Ct value was calculated by Spearman Rank correlation and Pearson’s product moment correlation.

For the 7SL-sRNA to be a suitable target for development of molecular diagnostics, there is a requirement that the sRNA is constitutively released into the bloodstream, rather than only under certain conditions such as cellular stress, as has recently been shown with, for example, the spliced leader RNA [36]. To investigate this, time courses lasting 3 days (72 hours) were carried out using *in vitro* cultures of both *T*. *brucei* (Lister 427) and *T*. *congolense* (IL3000). Cells were seeded at 5 × 10^4^ cells/mL (n = 2), and density was periodically counted by haemocytometer and supernatant samples were taken simultaneously for RT-qPCR analysis (Fig 3). By the first time-point, the small RNA was readily detected in both *T*. *brucei* (Fig 3A) and *T*. *congolense* (Fig 3B) supernatants (mean cell densities: *T*. *brucei*, 2.37 × 10^5^ cells/mL; *T*. *congolense*, 3.5 × 10^4^ cells/mL), as calculated relative to the zero hour time point. Furthermore, relative 7SL-sRNA levels appeared to increase correlating with cell density (*T*. *brucei*: Pearson = 0.7724, Spearman ρ = 0.9643; *T*. *congolense*: Pearson = 0.9353, Spearman ρ = 0.9643; Fig 3A). Taken together, these data indicate that the 7SL-sRNA is constitutively released by both species of parasite, and indeed, relative abundance of the sRNA can give an indication of cell density in parasite cultures. Interestingly, when 7SL-sRNA levels were corrected for cell number and directly compared, levels of 7SL-sRNA accumulated more rapidly in *T*. *congolense* than *T*. *brucei* cultures, suggesting there may be species-specific kinetics of extracellular production (Fig 3C).

### 7SL-sRNA is detected before parasitaemia is detectable by microscopy and during remission phase

**Fig 4:**
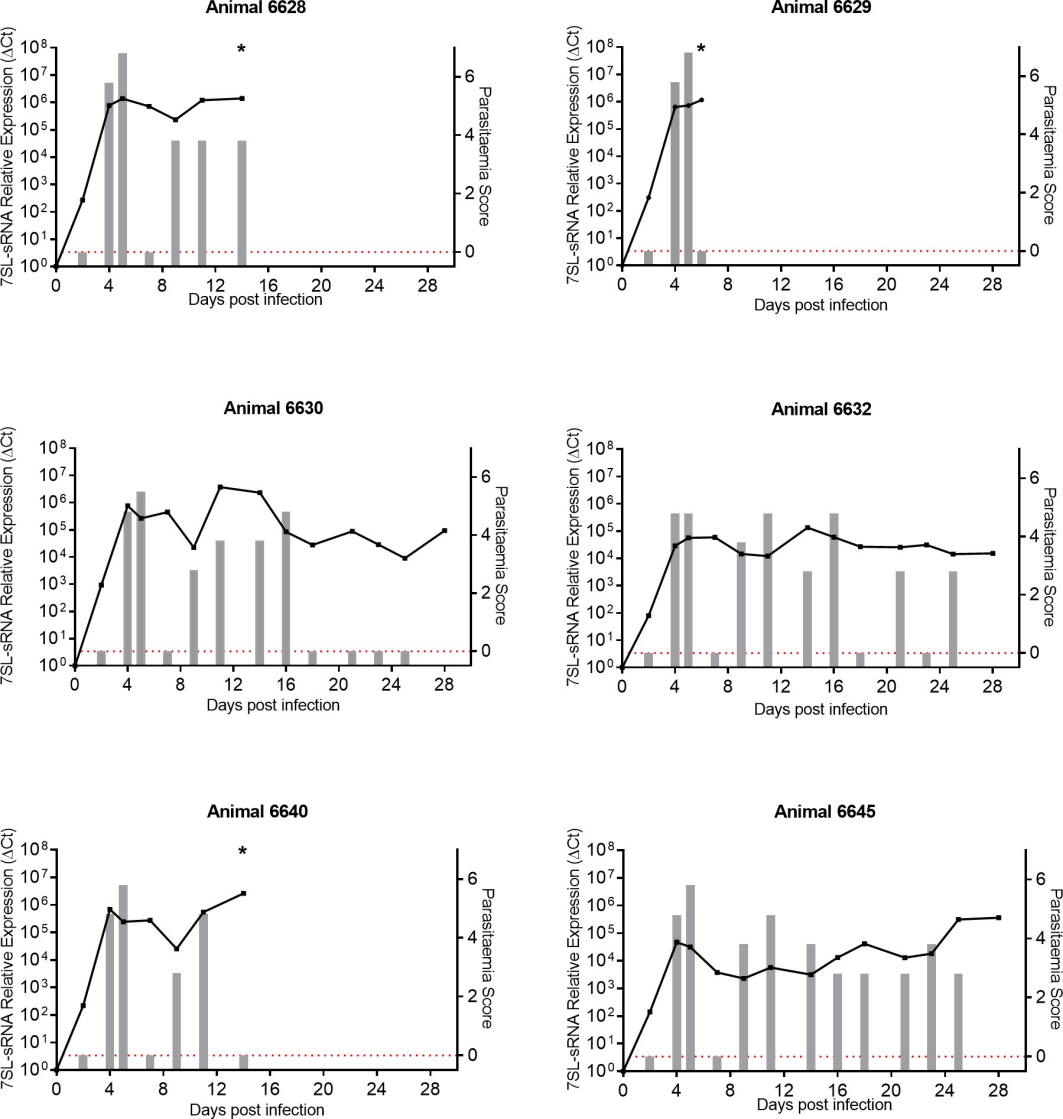
*In vivo* detection of *T*. *brucei*-specific 7SL-sRNA. Six calves (Holstein-Friesian male, approximately 4–6 months old) were infected with *T*. *brucei* (AnTat 1.1) and monitored for up to 28 days. Parasitaemia (right axis) was determined every 2 days on average, by microscopic detection of parasites; indicated by grey bars; approximate equivalent parasitaemia (parasites/mL): 1 = 1 x 10^2^; 2 = 1 x 10^3^; 3 = 1 x 10^4^; 4 = 1–5 x 10^5^; 5 = 5 x 10^5^–5 x 10^6^; 6 = >5 x 10^6^; grey bars measuring zero (red line) indicate where parasitaemia was measured but not detected, and no bar indicates that parasitaemia was not measured. Relative expression of 7SL-sRNA as measured by RT-qPCR (left axis) is shown by the black line graph, and was calculated by normalising to an uninfected serum control. Asterisk indicates euthanasia of an animal.

To further investigate the suitability of the 7SL-sRNA as a diagnostic for monitoring disease progression, serum samples were obtained from an *in vivo* study of six calves experimentally infected with 1 × 10^6^ *T*. *brucei* AnTat 1.1, which remained untreated for the duration of infection. The infection time courses ranged from six to 28 days depending on the severity of infection and day of euthanasia, and parasitaemia score was determined by microscopy approximately every two days (Fig 4). Total RNA was extracted from serum samples and analysed by RT-qPCR. The relative expression of 7SL-sRNA was calculated relative to the zero hour time point.

Parasites were typically detected in blood by microscopy after 4 days (Fig 4). In contrast, the 7SL-sRNA was detected by day 2, suggesting higher sensitivity compared to microscopy. Furthermore, following the first peak of parasitaemia, parasites became subpatent by microscopy, yet the 7SL-sRNA was still detectable at high levels during this time (animals 6630 and 6632; Fig 4).

Interestingly, data indicated that parasitaemia in animal 6630 remained undetectable by microscopy after day 16 (Fig 4), when no further parasites were detected until infections were terminated at day 28. However, 7SL-sRNA remained detectable, suggesting that this animal was suffering from a chronic stage of disease. Therefore, microscopy resulted in a false negative diagnosis but the RT-qPCR clearly remained sensitive, with a lower detection threshold than microscopy. However, the result could also indicate that the RNA is stable in the bloodstream and remains detectable after live parasites have been cleared. To investigate this further, we next focused our attention on animals undergoing treatment.

### Detection of 7SL small RNA accurately predicts active infection and parasite clearance

**Fig 5:**
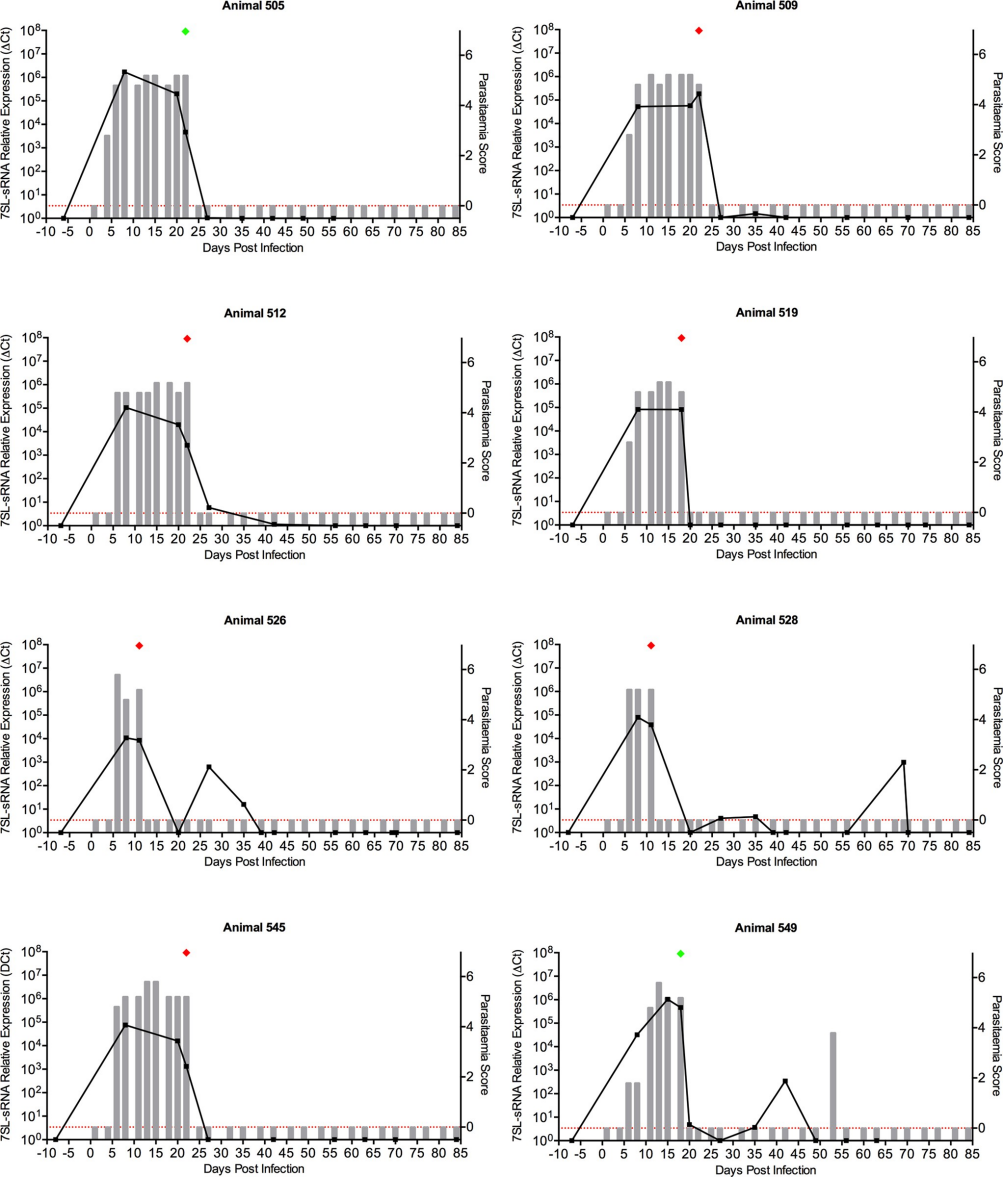
*In vivo* detection of *T*. *congolense*-specific 7SL-sRNA. Twenty-one cattle were challenged with *T*. *congolense* (KONT2/133), and subsequently divided into four groups depending on a treatment regimen with a candidate trypanocide. Of the 21, data from eight cattle are shown. Parasitaemia scores (right axis) were measured by microscopy every two to three days, indicated by grey bars; approximate equivalent parasitaemia (parasites/mL): 1 = 1 x 10^2^; 2 = 1 x 10^3^; 3 = 1 x 10^4^; 4 = 1–5 x 10^5^; 5 = 5 x 10^5^–5 x 10^6^; 6 = >5 x 10^6^; grey bars measuring zero (red line) indicate where parasitaemia was measured but not detected, and no bar indicates that parasitaemia was not measured. Plasma samples were obtained at longer intervals (approximately weekly) from which RNA was extracted; 7SL-RNA RT-qPCR results (left axis) are shown by the black line graph, and and were calculated by normalising to an uninfected serum control; green diamond indicates day when animal was treated with a rescue drug (isometamidium chloride or diminazene aceturate) and red diamond indicates when test drug was administered.

**Fig 6:**
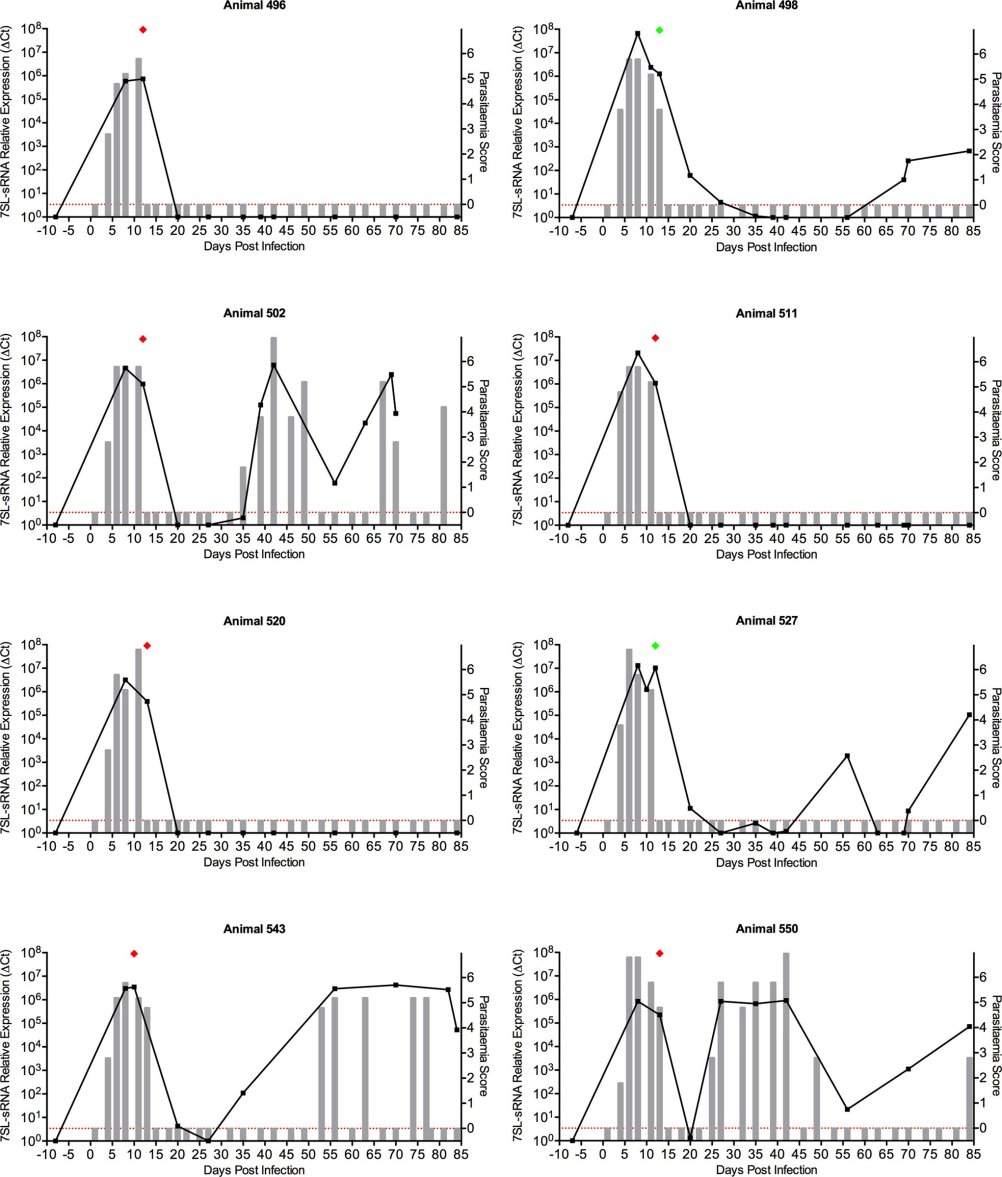
*In vivo* detection of *T*. *vivax*-specific 7SL-sRNA. Twenty-one cattle were challenged with *T*. *vivax* (STIB 719), and subsequently divided into four groups depending on a treatment regimen with a candidate trypanocide. Of the 21, data from eight cattle are shown. Parasitaemia scores (right axis) were measured by microscopy every two to three days, indicated by grey bars; approximate equivalent parasitaemia (parasites/mL): 1 = 1 x 10^2^; 2 = 1 x 10^3^; 3 = 1 x 10^4^; 4 = 1–5 x 10^5^; 5 = 5 x 10^5^–5 x 10^6^; 6 = >5 x 10^6^; grey bars measuring zero (red line) indicate where parasitaemia was measured but not detected, and no bar indicates that parasitaemia was not measured. Plasma samples were obtained at longer intervals (approximately weekly) from which RNA was extracted; 7SL-RNA RT-qPCR results (left axis) are shown by the black line graph, and and were calculated by normalising to an uninfected serum control; green diamond indicates day when animal was treated with a rescue drug (isometamidium chloride or diminazene aceturate) and red diamond indicates when test drug was administered.

Monitoring of disease progression is a vital aspect of treatment as well as for the development of optimised chemotherapeutics, which require their efficacy to be accurately measured during clinical trials. To this end, we used the 7SL-sRNA RT-qPCR assay on samples obtained from clinical trials performed on cattle experimentally infected with *T*. *congolense* (Fig 5) or *T*. *vivax* (Fig 6). The objectives of this study were primarily to test how the assay would compare with other traditional measurements of disease progression such as microscopy, and to evaluate whether the 7SL-sRNA remains present in the bloodstream when an infection is cleared by chemotherapy. Importantly, trial animals were monitored for 85 days, allowing long-term follow-up sampling and analysis, and assessment of the utility of the 7SL-sRNA as a marker of active infection (e.g. in the event of treatment failure).

In all 21 cattle infected with *T*. *congolense* (KONT 2/133) (Fig 5; full data in S1 Fig), an initial wave of parasitaemia was observed by microscopy after ~5 days. Whilst no plasma samples were available to test between day 0 and day 8, 7SL-sRNA was detected at the earliest post infection time-point available in all cattle. In these analyses data was normalised to a sample taken six to eight days preinfection. Upon experimental treatment of the cattle, there was a marked decrease in parasitaemia as determined by microscopy, which was mirrored by 7SL-sRNA detection assays carried out at the nearest time-points post-treatment (Fig 5). This observation was exemplified by animals 519 and 549, where plasma sampled just one day after treatment was available for testing and there was no detectable 7SL-sRNA signal, as well as there being no detectable trypanosomes by microscopy (Fig 5). Indeed, 7SL-sRNA was rarely detected after treatment for the duration of the trial.

Interestingly, in one case (animal 549), 7SL-sRNA was detected after 42 days, suggesting a relapse. Almost 15 days later, live trypanosomes were observed by microscopy, after which the infection was once again cleared (Fig 5). A similar phenomenon was observed in animals 526 and 528, where no parasitaemia was detected by microscopy, again suggesting relapse of infection, detectable by RT-qPCR but not by microscopy.

Importantly, these results indicate that 7SL-sRNA is short-lived *in vivo*, as successful drug treatment rapidly leads to the loss of signal, suggesting active infections are required to sustain the high abundance of the 7SL-sRNA. This further highlights the potential of 7SL-sRNA as a diagnostic for active trypanosome infections, rather than simply exposure such as is observed using antibody-based serological tests.

Samples from a second clinical trial involving 21 cattle experimentally infected with *T*. *vivax* (STIB 719) were also tested using the *T*. *vivax*-specific RT-qPCR assay (Fig 6; full data in S2 Fig). As with the *T*. *congolense* study, parasitaemia was measured every 2–3 days, and plasma samples were obtained more sporadically (approximately weekly) over a period of 85 days post-infection. Treatment was administered after peak parasitaemia was observed by microscopy, typically after ~14 days. For the *T*. *vivax* trials, rescue treatment was administered if the trial compound failed.

As demonstrated for *T*. *congolense*, 7SL-sRNA was detected by the first available time point, and once again mirrored parasitaemia observed by microscopy. In most cattle, *T*. *vivax* was cleared post-treatment (exemplified by animals 496, 511, 515, 520, 522, 532, 544, and 538), and the 7SL-sRNA signal was absent at the next sampling timepoint (typically 7–10 days later).

However, in several cases, the presence of 7SL-sRNA was detected in time points where no parasites were observed by microscopy, again highlighting the increased sensitivity exhibited by the RT-qPCR compared to microscopy. Indeed, in these cases, such as animals 498, 524 and 527, 7SL-sRNA appeared to indicate cyclical changes in parasitaemia commonly associated with trypanosome infections (Fig 6).

The above theory was further strengthened when investigating several animals that suffered from relapse of infection due to treatment failure, as confirmed by microscopy (in particular, animals 502, 543 and 550) (Fig 6). In animal 543, 7SL-sRNA plasma levels increased after day 30, without a corresponding increase in parasitaemia. Parasites were finally observed by microscopy on day 55, more than 3 weeks after detection of relapse by 7SL-sRNA.

Therefore, by RT-qPCR analysis of a highly abundant secretory/excretory small RNA, infection status was confirmed more accurately than by microscopy. Further analysis is required, ideally with time courses that include frequent sampling of host serum pre- and post-treatment, including at subtherapeutic doses, in order to accurately determine both the kinetics of decay of 7SL-RNA signal after successful treatment and the association between treatment failure, parasite dynamics and 7SL-RNA signal.

### 7SL-sRNA is detected directly from serum with one-step RT-qPCR assay

**Fig 7:**
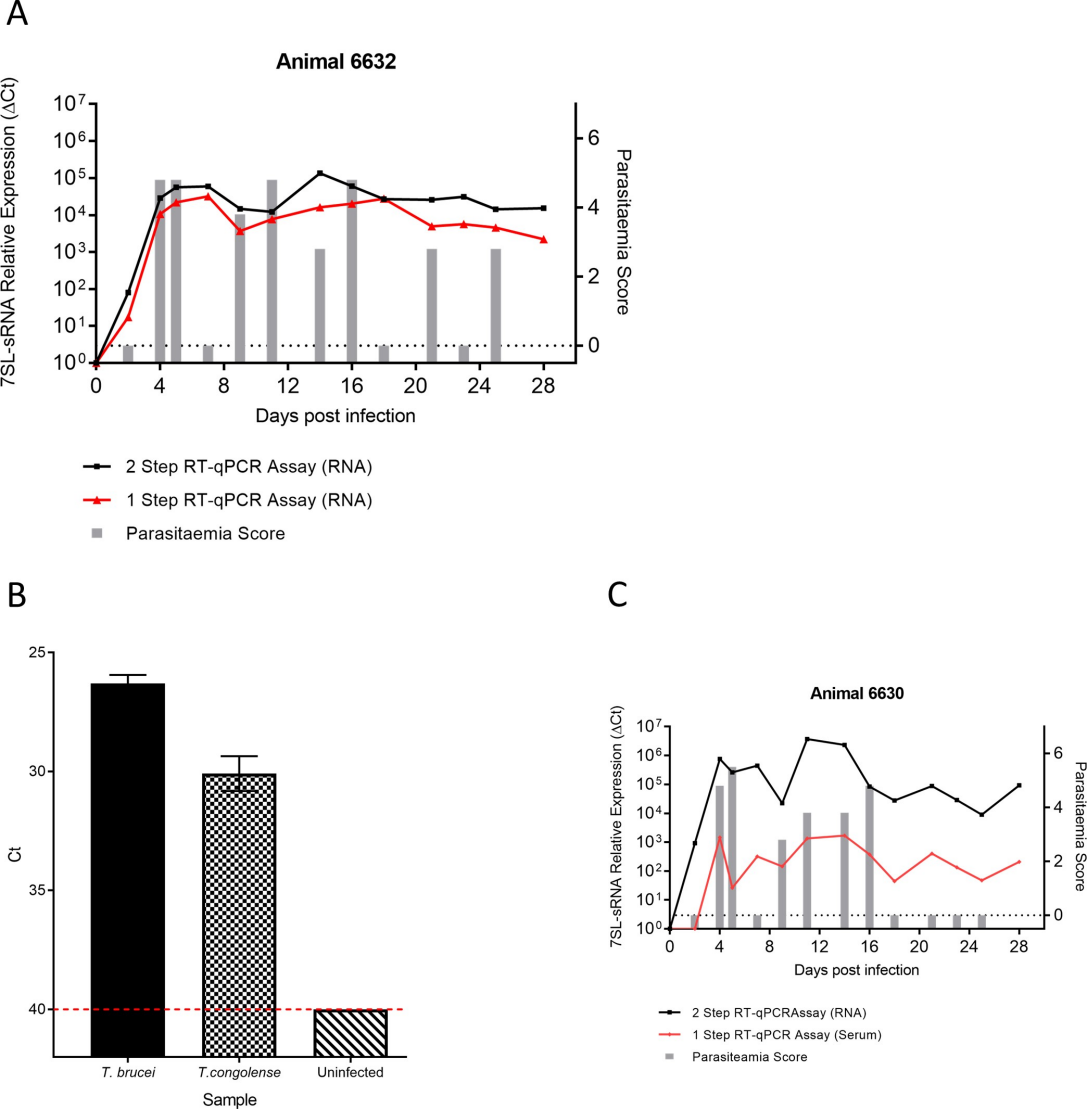
7SL-sRNA can be detected by directly from serum using a one-step RT-qPCR. A) Serum samples from a *T*. *brucei* infected cow (animal 6632, Fig 4) were directly applied to a one-step RT-qPCR reaction, and the results compared to the two-step RT-qPCR reaction and parasitaemia scoring by microscopy. In all cases the sRNA was detectable although expression levels were slightly decreased. Parasitaemia scores (right axis) are indicated by grey bars; grey bars measuring zero (red dashed line) indicate where parasitaemia was measured but not detected, and no bar indicates that parasitaemia was not measured. RT-qPCR results (left axis) are shown by the black line (two step RT-qPCR) and red line (one step RT-qPCR) graph, and and was calculated by normalising to an uninfected serum control. B) The use of serum as a direct substrate for the RT-qPCR was tested using 6 μL of serum, heat-treated at 65°C for 15mins, from both *T*. *brucei and T*. *congolense* infected animals, showing that the sRNA was detectable using this substrate (n = 2 per sample). C) The optimised one-step RT-qPCR assay was applied to serum samples from a *T*. *brucei*-infected cow (animal 6630, Fig 4) in order to assess performance when parasitaemia is undetectable for a substantial period. Similar to the two-step RT-qPCR carried out on RNA extracts, the 7SL-sRNA was still detectable when parasites were not detectable by microscopy for 10 days, as judged by microscopy. Parasitaemia scores (right axis) are indicated by grey bars; grey bars measuring zero (red dashed line) indicate where parasitaemia was measured but not detected, and no bar indicates that parasitaemia was not measured. RT-qPCR results (left axis) are shown by the black line (two step RT-qPCR) and red line (one step RT-qPCR) graph, and and was calculated by normalising to an uninfected serum control.

Whilst our data suggests that the 7SL-sRNA presents a realistic target for development of a molecular diagnostic for both HAT and AAT, in reality for diagnostic assays to be field-applicable in the settings in which both diseases occur, an assay requires several attributes: high on this list are two related aspects—low cost and minimal number of processing steps. A two-step RT-qPCR involving a lengthy RNA extraction protocol is therefore not desirable. We therefore investigated whether a one-step RT-qPCR would simplify the assay and if the RNA could be detected directly from serum samples without the requirement for RNA extraction (Fig 7).

Using RNA samples from *T*. *brucei* infected animal 6632 (Fig 4), we demonstrated that the one-step RT-qPCR detected 7SL-sRNA at every timepoint where the small RNA was detected by the two-step assay (Fig 7A). Additionally, serum or plasma samples from both *T*. *congolense* and *T*. *brucei*-infected cattle were assayed using one-step RT-qPCR reactions, which were performed on 6 μL serum/plasma in a 15 μL reaction. For both *T*. *brucei* and *T*. *congolense* (Fig 7B), 7SL-sRNA was readily detected. Finally, serum samples from a *T*. *brucei* in vivo infection time-course that included periods of patent and subpatent parasitaemia by microscopy (animal 6630) were re-tested using the single step assay (Fig 7C). Again the assay was able to clearly detect 7SL-sRNA, albeit with reduced sensitivity, during active infection even when infection was in remission and no parasitaemia could be detected by microscopy.

These results demonstrate that assays for 7SL-sRNA are species-specific, highly sensitive, and can be detected the RNA before the onset of parasitaemia as well as during periods where there is subpatent parasitaemia by microscopy. Moreover, the 7SL-sRNA can also be detected directly from serum using a one-step RT-qPCR assay.

## Discussion

To meet the challenges of both elimination of HAT and management of AAT, improved diagnostic techniques are crucial. Current methods of diagnosis are suboptimal, particularly for AAT, and this is hindering progress on reducing the disease burden [3, 4]. Traditionally diagnosis for both HAT and AAT relies upon microscopy—this depends on a minimum threshold of parasitaemia in order to accurately detect infection (even with parasite concentration methods), and as our data illustrates there are often long periods of subpatent parasitaemia in trypanosome infections that is then problematic for microscopy diagnosis. In addition, the traditional reliance upon the presence of parasites in venous blood does not take into account recent reports of extravascular reservoirs of the parasite, such as the skin [37, 38] and adipose tissue [39] (which also seems to correlate with periods of subpatency in peripheral blood). Several potentially useful diagnostic approaches are sensitive and specific (e.g. traditional PCR), but are expensive and not easily field-applicable, and DNA-based tests can face the issues of DNA still circulating post-treatment and the potential for easy cross-contamination. Finally, some antibody-based diagnostic tests are available, and are effective and field applicable, such as the CATT test for HAT and VerY Diag for AAT, but antibody tests have a challenge in differentiating between active infection and exposure (additionally the CATT test only detects *T*. *b*. *gambiense*—there is currently no field-applicable test available for *T*. *b*. *rhodesiense*). Therefore, sensitive and specific markers of active infection are still required for both HAT and AAT.

In this study we identified a species-specific small RNA excreted/secreted by all three AAT relevant species, as well as the main causative agent of HAT, *T*. *b*. *gambiense*, at physiologically relevant abundances both *in vivo* and *in vitro*. Crucially, the RNA, termed 7SL-sRNA, is indicative of active infection and is detected even when parasitaemia is below levels detectable by microscopy. Furthermore, successful drug treatment results in rapid loss of detectable 7SL-sRNA signal, thereby exhibiting a key characteristic of a marker that correlates with active infection.

Whilst this study was able to utilise samples from multiple experimental animal trials, human blood or CSF samples that would be suitable for testing could not be identified, although we hypothesise the 7SL-sRNA would also be present at similar levels in these types of clinical samples in human patients–we tested culture supernatant from *T*. *b*. *gambiense* (ELIANE) grown *in vitro*, and the level of signal was the same as observed with *T*. *b*. *brucei*. Developing a diagnostic for HAT that is able to detect both *T*. *b*. *gambiense* and *T*. *b*. *rhodesiense* would be greatly beneficial, as currently there is no molecular test that is widely used in the field for *T*. *b*. *rhodesiense* diagnosis [3].

Whilst the 7SL-sRNA is detectable using laboratory PCR machines, these assays are clearly not field applicable in their current state, and further work must be carried out to adapt the assays (e.g. to the loop mediated isothermal amplification (LAMP) platform; see below) to be field applicable. However, there have also been considerable efforts to exploit differential host small RNA levels as biomarkers of disease states in human medicine, in particular miRNAs in cancer, which has led to several other potentially field-applicable diagnostic techniques that could be adapted to detect 7SL-sRNA.

Several other technologies have the potential to be alternative platforms suitable for small RNA detection. In particular, the LAMP assay, which has been previously developed for all three livestock trypanosome species based on gDNA targets [13, 40, 41] and for which a test intended for field application was developed for HAT [14], has been shown to be suitable for application to small RNA as an assay substrate [42, 43] and therefore could potentially be optimised to develop a field-applicable 7SL-sRNA assay. Another recently developed method, Recombinase Polymerase Amplification (e.g. [44]), exhibits potential as an extremely sensitive detection method, even surpassing the aforementioned LAMP assay. This process requires a reaction consisting of a recombinase, a single-stranded DNA-binding protein and a strand-displacing polymerase to amplify the target [45]. Importantly, this method can be carried out at low temperatures, and amplification has been shown to proceed using just body heat [46]. By coupling this assay to a lateral flow device or a dipstick using biotinylated primers, the target can be visualised by eye, thereby bypassing the need for thermocycler or real-time fluorescence detection. Whilst RPA typically requires targets consisting of >30 bp, this technology has recently been adapted to the detection of miRNAs by ligating highly specific probes to the miRNA using a PBCV-1 ligase [47].

The discovery of a small RNA secreted/excreted by African trypanosomes at high abundance also raises interesting and potentially important biological questions. There is currently a great deal of interest in the ability of pathogens to communicate with each other and to manipulate host functions through the delivery of small RNAs [48]. Communication between trypanosomes during infection is required to regulate differentiation in a population density-dependant manner [49]. Trypanosome infection also has substantial effects on host cells, for example the ablation of B cells and consequent loss of immune memory through an as yet undefined ligand [50]. A small RNA, such as 7SL-sRNA could directly regulate gene expression, as is the case with miRNAs, or act as a signalling molecule, potentially triggering or inhibiting immunoregulatory pathways in host cells. Studies have demonstrated that small RNAs are often packaged, secreted and delivered to target cells via extracellular vesicles such as exosomes. The 7SL RNA has been detected in exosomes from other trypanosomatids including *Leishmania* spp. [51] and *Trypanosoma cruzi*, a closely related pathogen that causes Chagas’ disease in South America [52]. However, in these datasets, the significance of this finding and, indeed, whether the entire 7SL RNA or just a portion of it were observed, were not discussed. Studies are currently underway to determine whether 7SL-sRNA is released from the parasite in a vesicle or exists freely. Furthermore, it is yet to be determined whether 7SL-sRNA exists as part of a larger complex. Its stability in serum would suggest the RNA is somehow protected from RNase activity.

In addition to the potential biological role of 7SL-sRNA, how the small RNA is processed and released from trypanosomes is yet to be determined. The existence of a potential passenger strand could suggest a role for DICER in processing the larger 7SL RNA, as this type III endonuclease has previously been shown to process mammalian 7SL RNA [53]. Intriguingly, previous studies have noted the absence of a conserved eukaryotic SRP complex protein in a related trypanosomatid species. Instead, isolation of the 7SL RNA revealed a co-migratory tRNA-like molecule (sRNA-85 in *Leptomonas collosoma* [54], sRNA-76 in *T*. *brucei* [55]). The tRNA-like molecule has extensive and precise complementarity to the region of 7SL RNA that is processed to generate the small RNA. Finally, while our data is consistent with the 7SL-sRNA being actively processed and secreted/excreted, we cannot currently formally rule out that parasite cell death or membrane damage may be contributing to the 7SL-sRNA signal.

In summary, we have detected a trypanosome small RNA (7SL-sRNA), derived from the non-coding 7SL RNA of the SRP, which is excreted/secreted at high levels by *T*. *brucei*, *T*. *congolense* and *T*. *vivax* during infections. Species-specific RT-qPCR assays were developed, and we have shown that there is good correlation between 7SL-sRNA levels and parasite numbers, but importantly 7SL-sRNA can be detected both before patent parasitaemia and during phases of infection when parasitaemia becomes subpatent (both chronic infection and treatment failure), and critically the 7SL-sRNA signal decays rapidly after successful chemotherapy. Therefore, 7SL-sRNA represents a marker of active infection, and is a novel and viable target for the development of much needed diagnostics for both HAT and AAT, and may also provide insights into important host-pathogen interactions.

## Supporting information

S1 Table RNAseq analysis of small RNAs in the serum of *T*. *congolense*-infected cattle compared to uninfected serum and an *in vitro*-derived *T*. *congolense* cell pellet. (XLSX)

S2 Table Alignment statistics of the RNAseq data to the *T*. *congolense* (TriTrypDB, v9.0) and the *Bos Taurus* (UMD, v3.1) genomes using Novoalign (v3.02.12). (XLSX)

S1 Fig *In vivo* detection of *T*. *congolense* 7SL-sRNA. Twenty-one cattle were challenged with *T*. *congolense* KONT2/133, and subsequently divided into four groups depending on a treatment regimen with a candidate trypanocide. Data for the remaining 13 cattle are shown. Parasitaemia scores (right axis) were measured by microscopy every two to three days, indicated by grey bars; approximate equivalent parasitaemia (parasites/mL): 1 = 1 x 10^2^; 2 = 1 x 10^3^; 3 = 1 x 10^4^; 4 = 1–5 x 10^5^; 5 = 5 x 10^5^–5 x 10^6^; 6 = >5 x 10^6^; grey bars measuring zero (red line) indicate where parasitaemia was measured but not detected, and no bar indicates that parasitaemia was not measured. Plasma samples were obtained at longer intervals (approximately weekly) from which RNA was extracted; 7SL-RNA RT-qPCR results (left axis) are shown by the black line graph, and and were calculated by normalising to an uninfected serum control; green diamond indicates day when animal was treated with a rescue drug (isometamidium chloride or diminazene aceturate) and red diamond indicates when test drug was administered.
(PDF)

S2 Fig *In vivo* detection of *T*. *vivax* 7SL-sRNA. Twenty-one cattle were challenged with *T*. *vivax*, and subsequently divided into four groups depending on a treatment regimen with a candidate trypanocide. Data for the remaining 13 cattle are shown. Parasitaemia scores (right axis) were measured by microscopy every two to three days, indicated by grey bars; approximate equivalent parasitaemia (parasites/mL): 1 = 1 x 10^2^; 2 = 1 x 10^3^; 3 = 1 x 10^4^; 4 = 1–5 x 10^5^; 5 = 5 x 10^5^–5 x 10^6^; 6 = >5 x 10^6^; grey bars measuring zero (red line) indicate where parasitaemia was measured but not detected, and no bar indicates that parasitaemia was not measured. Plasma samples were obtained at longer intervals (approximately weekly) from which RNA was extracted; 7SL-RNA RT-qPCR results (left axis) are shown by the black line graph, and and were calculated by normalising to an uninfected serum control; green diamond indicates day when animal was treated with a rescue drug (isometamidium chloride or diminazene aceturate) and red diamond indicates when test drug was administered.
(PDF)
