# Supplementary figures and images for: Parasite specific 7SL-derived small RNA is an effective target for diagnosis of active trypanosomiasis infection
Source: PLoS Negl Trop Dis. 2019 Feb 19;13(2):e0007189. doi: 10.1371/journal.pntd.0007189 (PMC6413958; doi:10.1371/journal.pntd.0007189)

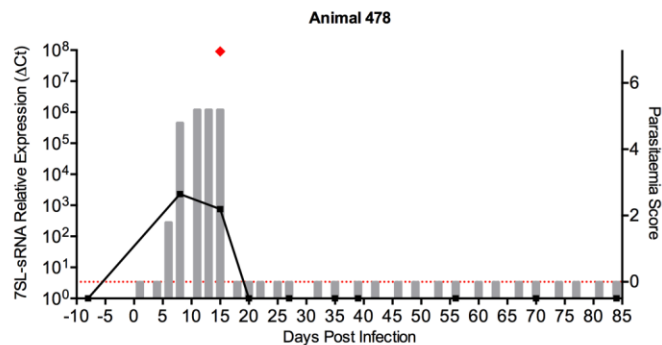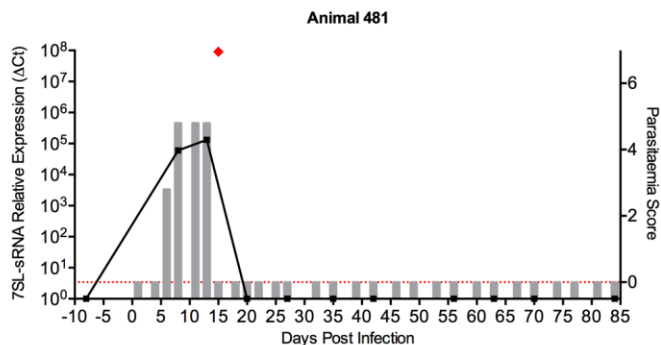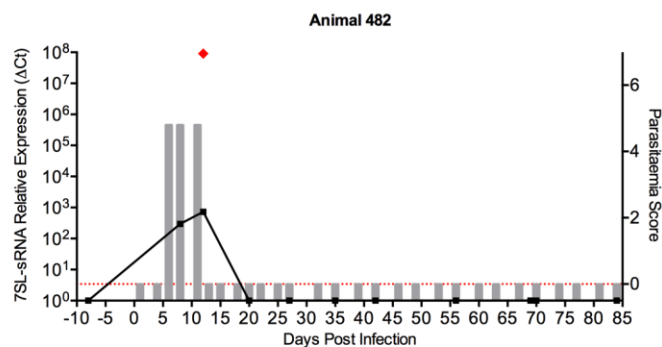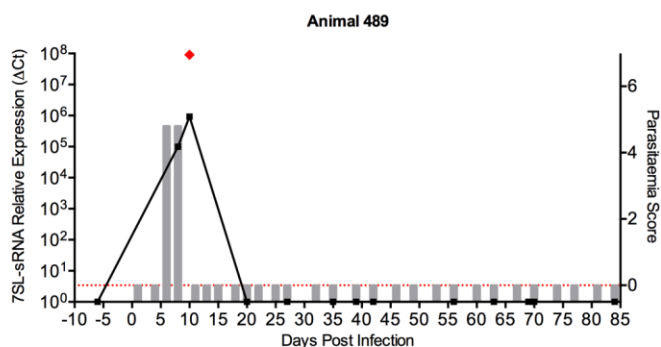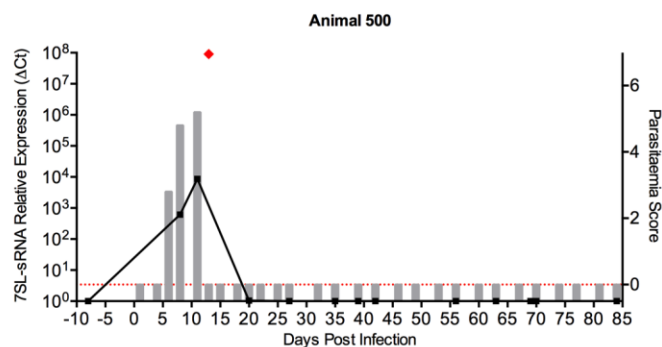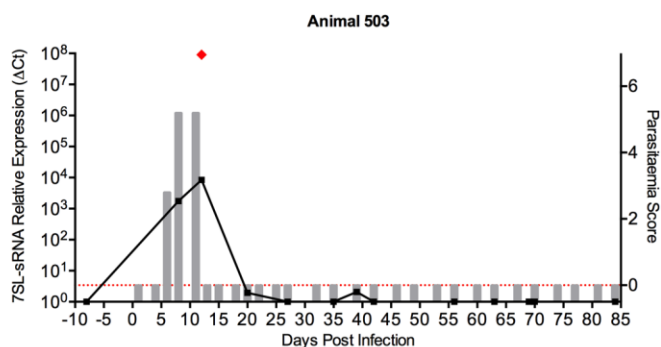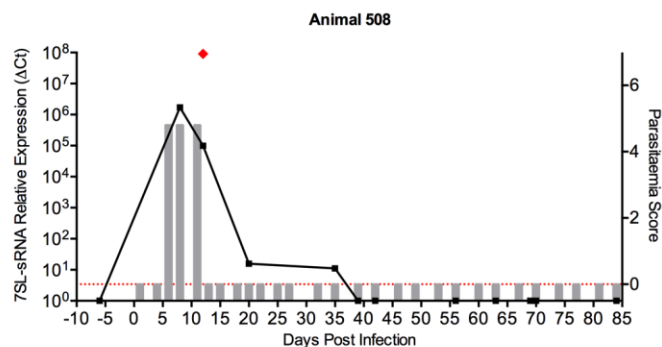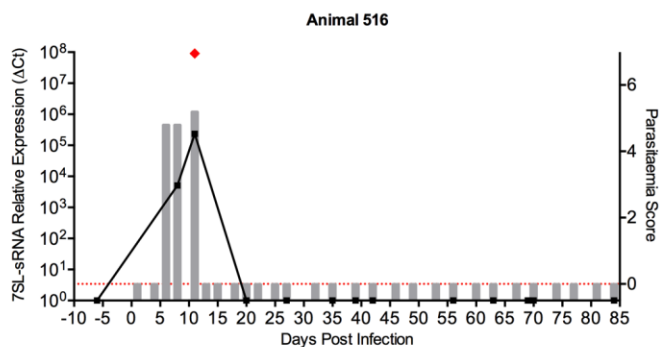

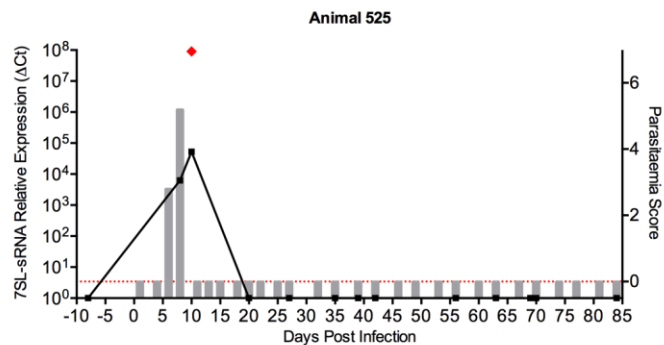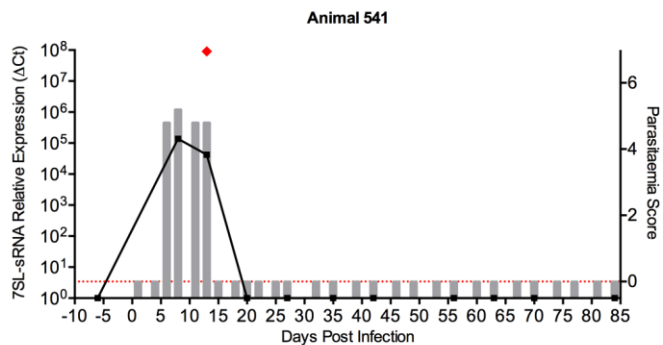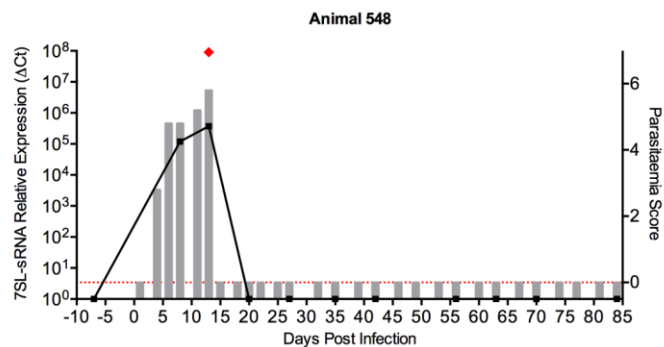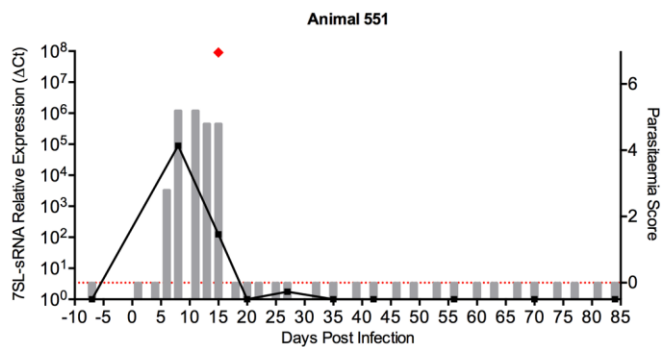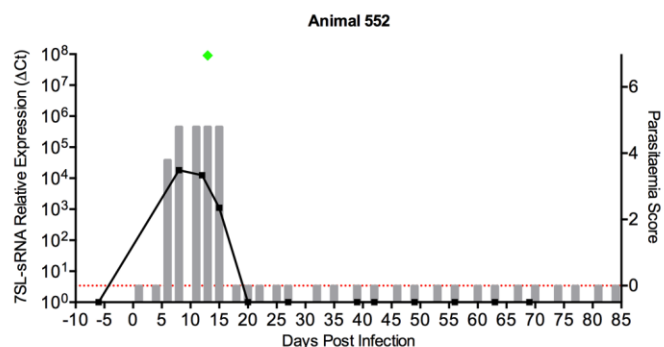

Supplement: S1 Fig — Twenty-one cattle were challenged with T. congolense KONT2/133, and subsequently divided into four groups depending on a treatment regimen with a candidate trypanocide. Data for the remaining 13 cattle are shown. Parasitaemia scores (right axis) were measured by microscopy every two to three days, indicated by grey bars; approximate equivalent parasitaemia (parasites/mL): 1 = 1 x 10^2^; 2 = 1 x 10^3^; 3 = 1 x 10^4^; 4 = 1–5 x 10^5^; 5 = 5 x 10^5^–5 x 10^6^; 6 = >5 x 10^6^; grey bars measuring zero (red line) indicate where parasitaemia was measured but not detected, and no bar indicates that parasitaemia was not measured. Plasma samples were obtained at longer intervals (approximately weekly) from which RNA was extracted; 7SL-RNA RT-qPCR results (left axis) are shown by the black line graph, and and were calculated by normalising to an uninfected serum control; green diamond indicates day when animal was treated with a rescue drug (isometamidium chloride or diminazene aceturate) and red diamond indicates when test drug was administered. (PDF) [file pntd.0007189.s003.pdf]

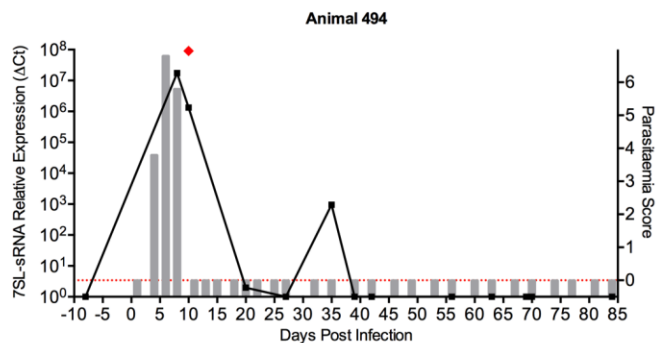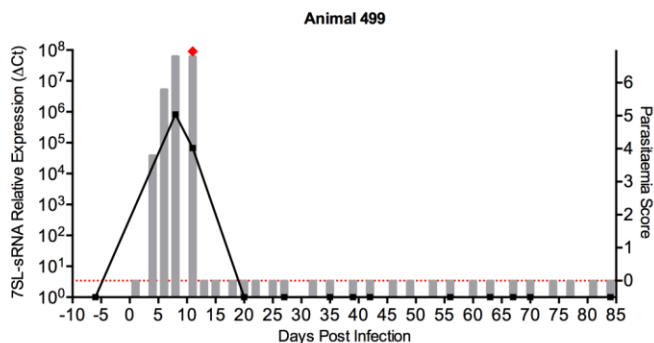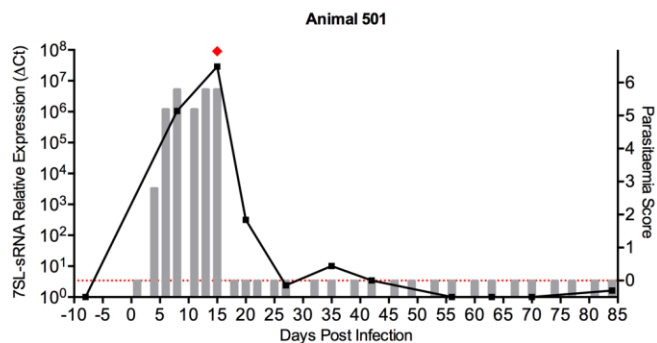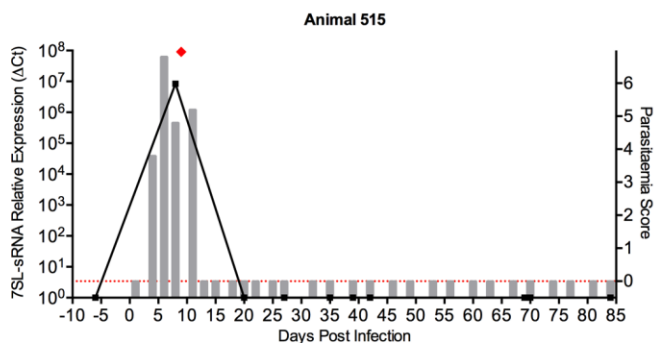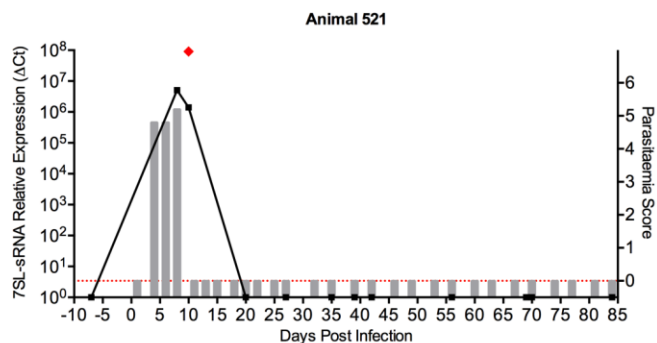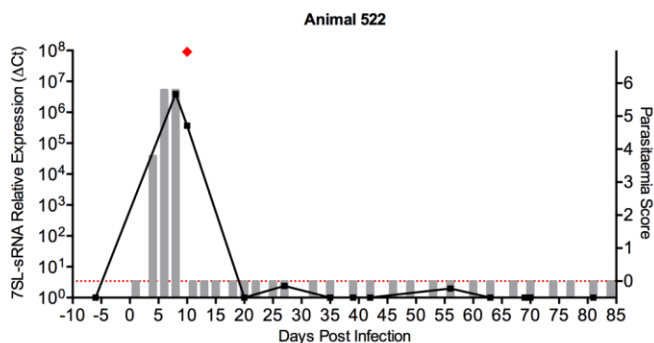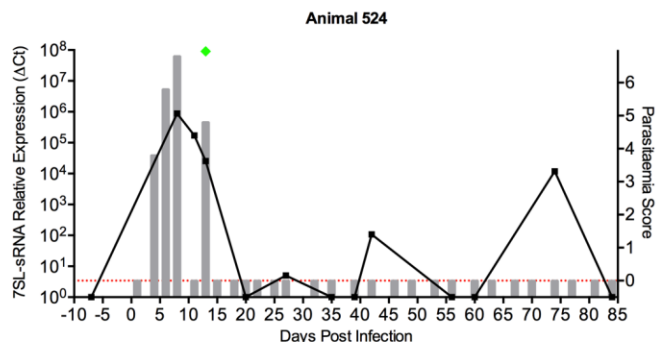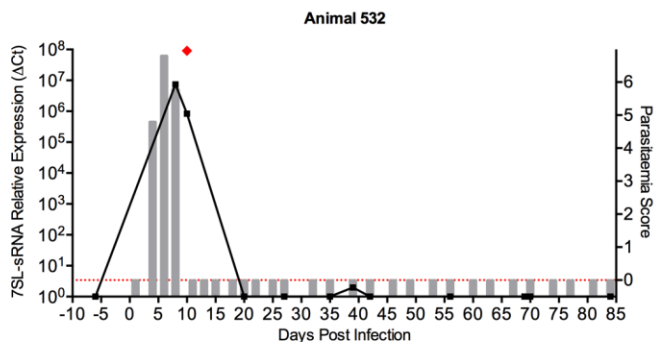

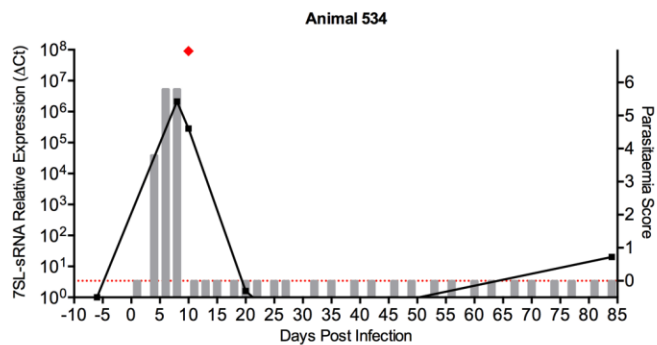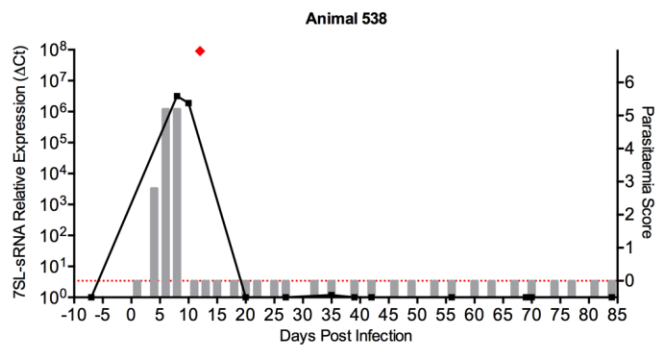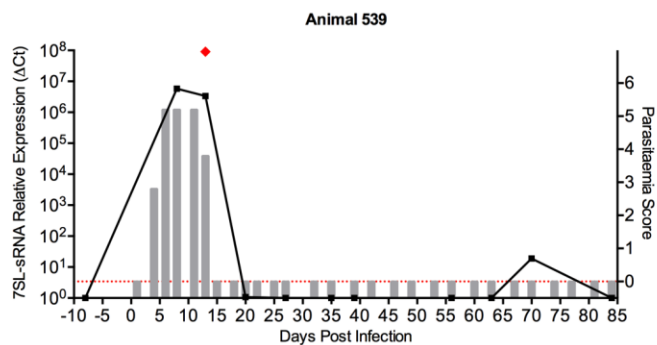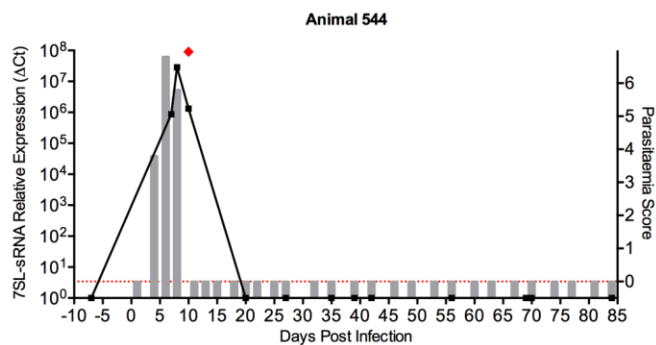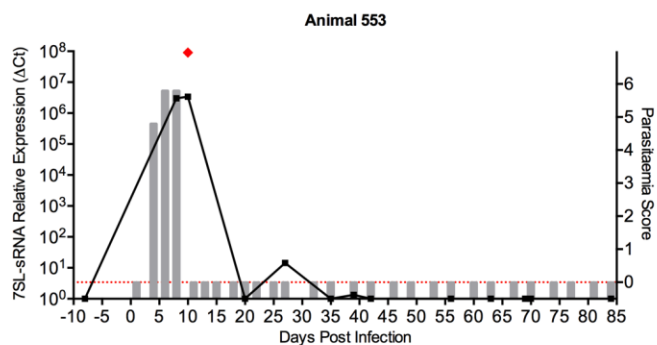

Supplement: S2 Fig — Twenty-one cattle were challenged with T. vivax, and subsequently divided into four groups depending on a treatment regimen with a candidate trypanocide. Data for the remaining 13 cattle are shown. Parasitaemia scores (right axis) were measured by microscopy every two to three days, indicated by grey bars; approximate equivalent parasitaemia (parasites/mL): 1 = 1 x 10^2^; 2 = 1 x 10^3^; 3 = 1 x 10^4^; 4 = 1–5 x 10^5^; 5 = 5 x 10^5^–5 x 10^6^; 6 = >5 x 10^6^; grey bars measuring zero (red line) indicate where parasitaemia was measured but not detected, and no bar indicates that parasitaemia was not measured. Plasma samples were obtained at longer intervals (approximately weekly) from which RNA was extracted; 7SL-RNA RT-qPCR results (left axis) are shown by the black line graph, and and were calculated by normalising to an uninfected serum control; green diamond indicates day when animal was treated with a rescue drug (isometamidium chloride or diminazene aceturate) and red diamond indicates when test drug was administered. (PDF) [file pntd.0007189.s004.pdf]
